# 2-[(Pyridin-2-yl)amino]­pyridinium 2,4,6-tri­nitro­phenolate

**DOI:** 10.1107/S1600536814012835

**Published:** 2014-06-14

**Authors:** Kateryna A. Ohui, Irina A. Golenya, Nadezhda A. Bokach

**Affiliations:** a Department of Chemistry, Taras Shevchenko National University of Kyiv, Volodymyrska 64/13, 01601 Kyiv, Ukraine; b Department of Chemistry, Saint Petersburg State University, Universitetsky Pr. 26, 198504 Stary Petergof, Russian Federation

## Abstract

In the cation of the title salt, C_10_H_10_N_3_^+^·C_6_H_2_N_3_O_7_^−^, the pyridine and pyridinium rings are linked by an intra­molecular N—H⋯N hydrogen bond and are approximately coplanar, with a dihedral angle between their planes of 4.24 (6)°. In the crystal, the cations and anions are linked through N—H⋯O hydrogen bonds, forming supra­molecular chains propagating along the *c*-axis direction. π–π stacking is observed between neighbouring chains, the centroid–centroid distances being 3.7638 (11) (between pyridinium rings) and 3.5331 (11) Å (between benzene rings).

## Related literature   

For uses of picric acid and picrates, see: Shriner *et al.* (1980 ▶); In *et al.* (1997 ▶); Zaderenko *et al.* (1997 ▶). For related structures, see: Fritsky *et al.* (2006 ▶); Moroz *et al.* (2012 ▶); Penkova *et al.* (2009 ▶); Golenya *et al.* (2012 ▶).
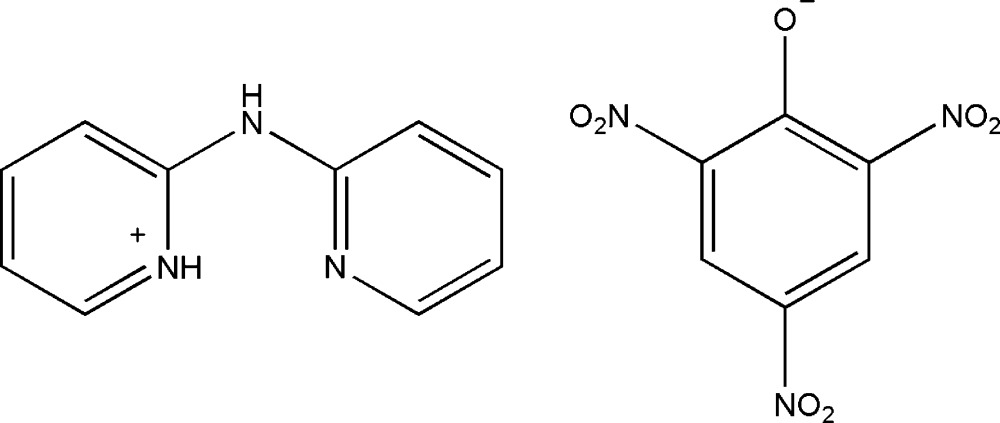


## Experimental   

### 

#### Crystal data  

C_10_H_10_N_3_^+^·C_6_H_2_N_3_O_7_^−^
*M* *_r_* = 400.32
Monoclinic, 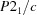

*a* = 9.1807 (8) Å
*b* = 14.5892 (12) Å
*c* = 13.1649 (10) Å
β = 108.925 (2)°
*V* = 1668.0 (2) Å^3^
*Z* = 4
Mo *K*α radiation
μ = 0.13 mm^−1^
*T* = 100 K
0.25 × 0.21 × 0.18 mm

#### Data collection  

Bruker Kappa APEXII CCD diffractometer
Absorption correction: multi-scan (*SADABS*; Bruker, 2002 ▶) *T*_min_ = 0.974, *T*_max_ = 0.983
10460 measured reflections
3253 independent reflections
2674 reflections with *I* > 2σ(*I*)
*R*_int_ = 0.029

#### Refinement  

*R*[*F* ^2^ > 2σ(*F* ^2^)] = 0.043
*wR*(*F* ^2^) = 0.102
*S* = 1.03
3253 reflections
270 parameters
H atoms treated by a mixture of independent and constrained refinement
Δρ_max_ = 0.59 e Å^−3^
Δρ_min_ = −0.55 e Å^−3^

### 

Data collection: *APEX2* (Bruker, 2007 ▶); cell refinement: *SAINT* (Bruker, 2007 ▶); data reduction: *SAINT* (Bruker, 2007 ▶); program(s) used to solve structure: *SHELXS97* (Sheldrick, 2008 ▶); program(s) used to refine structure: *SHELXL97* (Sheldrick, 2008 ▶); molecular graphics: *DIAMOND* (Brandenburg, 2009 ▶); software used to prepare material for publication: *SHELXL97* (Sheldrick, 2008 ▶).

## Supplementary Material

Crystal structure: contains datablock(s) I, New_Global_Publ_Block. DOI: 10.1107/S1600536814012835/xu5794sup1.cif

Structure factors: contains datablock(s) I. DOI: 10.1107/S1600536814012835/xu5794Isup2.hkl

Supporting information file. DOI: 10.1107/S1600536814012835/xu5794Isup3.cml

CCDC reference: 1006378

Additional supporting information:  crystallographic information; 3D view; checkCIF report

## Figures and Tables

**Table 1:** Hydrogen-bond geometry (Å, °)

*D*—H⋯*A*	*D*—H	H⋯*A*	*D*⋯*A*	*D*—H⋯*A*
N1—H1*N*⋯N3	0.92 (2)	1.86 (2)	2.618 (2)	139 (2)
N1—H1*N*⋯O4^i^	0.92 (2)	2.45 (2)	3.056 (2)	123.8 (17)
N2—H2*N*⋯O1^ii^	0.87 (2)	1.97 (2)	2.756 (2)	149 (2)
N2—H2*N*⋯O2^ii^	0.87 (2)	2.42 (2)	3.114 (2)	136.6 (18)

Symmetry codes: (i) 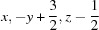; (ii) 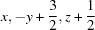.

**Fig. 1.**
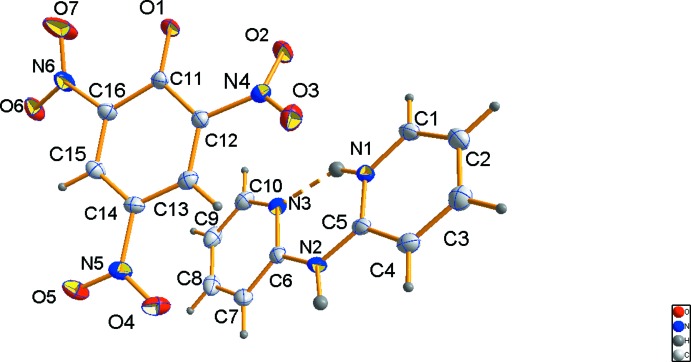
The molecular structure of the title compound, with atom labelling. Displacement ellipsoids are drawn at the 50% probability level.

**Fig. 2.**
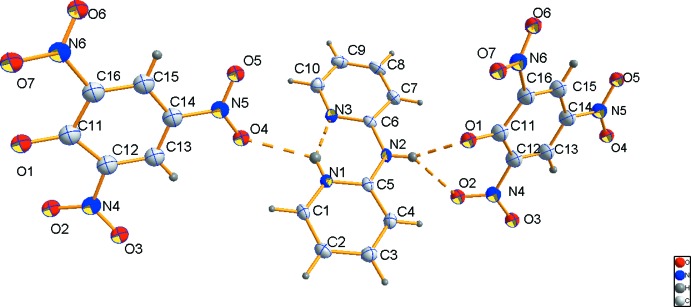
A view of the one-dimensional chain formed via hydrogen bonds.

**Fig. 3.**
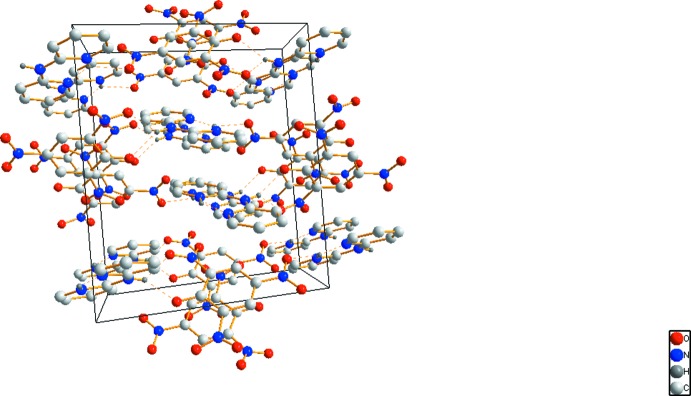
A packing diagram of the title compound. Hydrogen bonds are indicated by dashed lines. H atoms not involved in hydrogen bonding have been omitted for clarity.

## supplementary crystallographic information

### S0.1. Synthesis and crystallization

The 2,2'-di­pyridyl­amine (1.71 g, 0.01 mol) was added to picric acid (2.29 g, 0.01 mol) dissolved in ethanol (50ml). The obtained mixture was stirred for 24 hours, then this solution was left at room temperature for crystallization in the air. The crystals grown within 24 hours were separated, washed with ethanol and air-dried.

### S0.2. Refinement

The NH hydrogen atoms were located from the difference Fourier map and refined isotropically. Other hydrogen atoms were positioned geometrically and were also constrained to ride on their parent atoms, with C—H = 0.95 Å, and U_iso_(H) = 1.2U_eq_(C).

### S1. Results and discussion

Picric acid is a common reagent widely used for isolation in crystalline state of various organic compounds, in particular, amines and alkaloids (Shriner *et al.*, 1980). In laboratorial practice it is also used in analysis of amines and polycyclic hydro­carbons (In *et al.*, 1997; Zaderenko *et al.*, 1997).

In the present communication we report the molecular structure of 2-(2-pyridyl­amino)­pyridinium picrate. The crystal structure of the title compound C_10_H_10_N_3_^+^·C_6_H_2_N_3_O_7_^-^ is ionic and consists of the protonated cation C_10_H_10_N_3_^+^ and the deprotonated picrate anion C_6_H_2_N_3_O_7_^-^. The cation is approximately planar. The dihedral angle between two heterocyclic rings is 4.24 (6)°. The picrate anion is not planar: the three nitro-groups [N4—O2—O3], [N5—O4—O5], [N6—O6—O7] are twisted with respect to the benzene ring, the dihedral angles between their square - least planes are 26.8 (2)°, 4.5 (1)°, 23.0 (3)°. The C—N and C—C bond lengths in the pyridine rings are normal for 2-substituted pyridine derivatives (Fritsky *et al.*, 2006; Moroz *et al.*, 2012; Penkova *et al.*, 2009; Golenya *et al.*, 2012).

The 2-(2-pyridyl­amino)­pyridinium cation and the picrate anion are connected to each other by the existence of N—H···O and N—H···N hydrogen-bonding inter­actions and form one-dimensional chains. The protonated NH pyridinium group of the 2-(2-pyridyl­amino)­pyridinium cation is involved in a bifurcated inter­molecular hydrogen bond with the oxygen atom of the nitro-group and the pyr­idyne nitro­gen atom. The second of these H-bonds is an intra­molecular. The amine oxygen atom of the 2-(2-pyridyl­amino)­pyridinium cation also forms bifurcate N2—H2···O1 and N2—H2···O2 hydrogen bonds with the oxygen atom of the nitro-group and with the oxygen atom of the deprotonated hydroxo-group of the picrate anion.

### Crystal data

**Table:**

C_10_H_10_N_3_^+^·C_6_H_2_N_3_O_7_^−^	*F*(000) = 824
*M**_r_* = 400.32	*D*_x_ = 1.594 Mg m^−^^3^
Monoclinic, *P*2_1_/*c*	Mo *K*α radiation, λ = 0.71073 Å
Hall symbol: -P 2ybc	Cell parameters from 3858 reflections
*a* = 9.1807 (8) Å	θ = 2.4–29.9°
*b* = 14.5892 (12) Å	µ = 0.13 mm^−^^1^
*c* = 13.1649 (10) Å	*T* = 100 K
β = 108.925 (2)°	Block, yellow
*V* = 1668.0 (2) Å^3^	0.25 × 0.21 × 0.18 mm
*Z* = 4	

### Data collection

**Table:**

Bruker Kappa APEXII CCD diffractometer	3253 independent reflections
Radiation source: fine-focus sealed tube	2674 reflections with *I* > 2σ(*I*)
Horizontally mounted graphite crystal monochromator	*R*_int_ = 0.029
Detector resolution: 16 pixels mm^-1^	θ_max_ = 26.0°, θ_min_ = 2.2°
φ scans and ω scans with κ offset	*h* = −11→11
Absorption correction: multi-scan (*SADABS*; Bruker, 2002)	*k* = −17→16
*T*_min_ = 0.974, *T*_max_ = 0.983	*l* = −16→16
10460 measured reflections	

### Refinement

**Table:**

Refinement on *F*^2^	Primary atom site location: structure-invariant direct methods
Least-squares matrix: full	Secondary atom site location: difference Fourier map
*R*[*F*^2^ > 2σ(*F*^2^)] = 0.043	Hydrogen site location: inferred from neighbouring sites
*wR*(*F*^2^) = 0.102	H atoms treated by a mixture of independent and constrained refinement
*S* = 1.03	*w* = 1/[σ^2^(*F*_o_^2^) + (0.0416*P*)^2^ + 1.2391*P*] where *P* = (*F*_o_^2^ + 2*F*_c_^2^)/3
3253 reflections	(Δ/σ)_max_ < 0.001
270 parameters	Δρ_max_ = 0.59 e Å^−^^3^
0 restraints	Δρ_min_ = −0.55 e Å^−^^3^

### Special details

**Table:**

Geometry. All esds (except the esd in the dihedral angle between two l.s. planes) are estimated using the full covariance matrix. The cell esds are taken into account individually in the estimation of esds in distances, angles and torsion angles; correlations between esds in cell parameters are only used when they are defined by crystal symmetry. An approximate (isotropic) treatment of cell esds is used for estimating esds involving l.s. planes.
Refinement. Refinement of F^2^ against ALL reflections. The weighted R-factor wR and goodness of fit S are based on F^2^, conventional R-factors R are based on F, with F set to zero for negative F^2^. The threshold expression of F^2^ > 2sigma(F^2^) is used only for calculating R-factors(gt) etc. and is not relevant to the choice of reflections for refinement. R-factors based on F^2^ are statistically about twice as large as those based on F, and R- factors based on ALL data will be even larger.

### Fractional atomic coordinates and isotropic or equivalent isotropic displacement parameters (Å^2^)

**Table:**

	*x*	*y*	*z*	*U*_iso_*/*U*_eq_	
O1	0.10222 (15)	0.54091 (9)	−0.17771 (10)	0.0236 (3)	
O2	−0.06688 (18)	0.69273 (10)	−0.18938 (11)	0.0323 (4)	
O3	−0.16715 (15)	0.69936 (9)	−0.06137 (11)	0.0259 (3)	
O4	0.15975 (16)	0.63990 (10)	0.29479 (10)	0.0279 (3)	
O5	0.35245 (15)	0.54601 (10)	0.32327 (10)	0.0282 (3)	
O6	0.46259 (16)	0.40445 (10)	0.03187 (11)	0.0289 (3)	
O7	0.2807 (2)	0.39426 (15)	−0.11952 (13)	0.0702 (7)	
N1	−0.01574 (18)	0.88497 (10)	−0.04563 (12)	0.0176 (3)	
H1N	0.079 (3)	0.8656 (16)	−0.0451 (18)	0.036 (6)*	
N2	0.08719 (18)	0.86091 (11)	0.14029 (12)	0.0199 (3)	
H2N	0.075 (2)	0.8730 (15)	0.2019 (18)	0.027 (6)*	
N3	0.26368 (18)	0.82539 (11)	0.05248 (12)	0.0213 (4)	
N4	−0.06696 (18)	0.67256 (11)	−0.09846 (12)	0.0212 (3)	
N5	0.24186 (18)	0.58829 (11)	0.26247 (12)	0.0210 (4)	
N6	0.3395 (2)	0.42975 (12)	−0.03160 (13)	0.0288 (4)	
C1	−0.1325 (2)	0.90766 (13)	−0.13575 (14)	0.0206 (4)	
H1	−0.1166	0.9064	−0.2035	0.025*	
C2	−0.2720 (2)	0.93213 (13)	−0.12947 (15)	0.0232 (4)	
H2	−0.3541	0.9481	−0.1923	0.028*	
C3	−0.2925 (2)	0.93344 (13)	−0.02863 (15)	0.0238 (4)	
H3	−0.3894	0.9507	−0.0230	0.029*	
C4	−0.1746 (2)	0.91021 (13)	0.06177 (15)	0.0216 (4)	
H4	−0.1889	0.9113	0.1300	0.026*	
C5	−0.0325 (2)	0.88481 (12)	0.05260 (14)	0.0173 (4)	
C6	0.2352 (2)	0.83475 (12)	0.14498 (15)	0.0192 (4)	
C7	0.3446 (2)	0.81826 (13)	0.24528 (15)	0.0242 (4)	
H7	0.3202	0.8258	0.3096	0.029*	
C8	0.4886 (2)	0.79074 (14)	0.24768 (17)	0.0292 (5)	
H8	0.5662	0.7791	0.3144	0.035*	
C9	0.5205 (2)	0.77997 (14)	0.15200 (18)	0.0304 (5)	
H9	0.6193	0.7604	0.1522	0.036*	
C10	0.4057 (2)	0.79824 (14)	0.05736 (17)	0.0278 (5)	
H10	0.4277	0.7914	−0.0079	0.033*	
C11	0.1351 (2)	0.55224 (12)	−0.07926 (14)	0.0170 (4)	
C12	0.0569 (2)	0.61648 (12)	−0.02980 (14)	0.0171 (4)	
C13	0.0877 (2)	0.62673 (12)	0.07851 (14)	0.0176 (4)	
H13	0.0282	0.6674	0.1055	0.021*	
C14	0.2068 (2)	0.57704 (12)	0.14803 (14)	0.0180 (4)	
C15	0.2908 (2)	0.51464 (13)	0.10980 (14)	0.0188 (4)	
H15	0.3731	0.4814	0.1584	0.023*	
C16	0.2538 (2)	0.50156 (12)	0.00165 (14)	0.0190 (4)	

### Atomic displacement parameters (Å^2^)

**Table:**

	*U*^11^	*U*^22^	*U*^33^	*U*^12^	*U*^13^	*U*^23^
O1	0.0346 (8)	0.0227 (7)	0.0129 (6)	0.0057 (6)	0.0068 (6)	0.0021 (6)
O2	0.0471 (9)	0.0303 (8)	0.0171 (7)	0.0138 (7)	0.0070 (6)	0.0061 (6)
O3	0.0236 (7)	0.0218 (7)	0.0316 (8)	0.0031 (6)	0.0082 (6)	−0.0005 (6)
O4	0.0306 (8)	0.0354 (8)	0.0201 (7)	−0.0002 (6)	0.0116 (6)	−0.0062 (6)
O5	0.0250 (7)	0.0407 (9)	0.0158 (7)	0.0015 (6)	0.0021 (6)	0.0036 (6)
O6	0.0269 (8)	0.0336 (8)	0.0272 (7)	0.0120 (6)	0.0102 (6)	0.0095 (6)
O7	0.0931 (15)	0.0786 (14)	0.0221 (9)	0.0595 (12)	−0.0045 (9)	−0.0153 (9)
N1	0.0190 (8)	0.0170 (8)	0.0162 (8)	−0.0009 (6)	0.0052 (6)	−0.0003 (6)
N2	0.0224 (8)	0.0240 (8)	0.0132 (8)	0.0013 (7)	0.0056 (6)	−0.0013 (7)
N3	0.0222 (8)	0.0207 (8)	0.0215 (8)	−0.0001 (7)	0.0076 (7)	−0.0024 (7)
N4	0.0242 (8)	0.0166 (8)	0.0195 (8)	−0.0003 (7)	0.0026 (7)	−0.0029 (7)
N5	0.0212 (8)	0.0256 (8)	0.0161 (8)	−0.0062 (7)	0.0060 (7)	−0.0018 (7)
N6	0.0354 (10)	0.0328 (10)	0.0180 (8)	0.0142 (8)	0.0084 (7)	0.0023 (8)
C1	0.0252 (10)	0.0201 (9)	0.0147 (9)	−0.0021 (8)	0.0041 (7)	0.0006 (8)
C2	0.0225 (10)	0.0226 (10)	0.0213 (10)	0.0005 (8)	0.0025 (8)	0.0025 (8)
C3	0.0215 (10)	0.0228 (10)	0.0271 (10)	0.0009 (8)	0.0079 (8)	−0.0010 (8)
C4	0.0255 (10)	0.0215 (10)	0.0191 (9)	−0.0008 (8)	0.0092 (8)	−0.0023 (8)
C5	0.0228 (10)	0.0128 (8)	0.0155 (9)	−0.0028 (7)	0.0051 (7)	−0.0021 (7)
C6	0.0207 (9)	0.0141 (9)	0.0203 (9)	−0.0014 (7)	0.0034 (7)	−0.0025 (7)
C7	0.0271 (10)	0.0220 (10)	0.0197 (9)	−0.0001 (8)	0.0023 (8)	−0.0029 (8)
C8	0.0252 (11)	0.0229 (10)	0.0302 (11)	0.0018 (8)	−0.0036 (8)	−0.0031 (9)
C9	0.0208 (10)	0.0276 (11)	0.0399 (12)	0.0017 (8)	0.0059 (9)	−0.0076 (10)
C10	0.0273 (11)	0.0266 (11)	0.0316 (11)	0.0019 (9)	0.0124 (9)	−0.0043 (9)
C11	0.0199 (9)	0.0150 (9)	0.0154 (9)	−0.0033 (7)	0.0049 (7)	0.0012 (7)
C12	0.0171 (9)	0.0147 (9)	0.0178 (9)	−0.0023 (7)	0.0032 (7)	0.0002 (7)
C13	0.0182 (9)	0.0160 (9)	0.0196 (9)	−0.0043 (7)	0.0074 (7)	−0.0022 (7)
C14	0.0192 (9)	0.0209 (9)	0.0140 (9)	−0.0046 (7)	0.0057 (7)	−0.0019 (7)
C15	0.0156 (9)	0.0222 (9)	0.0172 (9)	−0.0014 (7)	0.0034 (7)	0.0022 (8)
C16	0.0200 (9)	0.0208 (9)	0.0173 (9)	0.0001 (8)	0.0074 (8)	0.0001 (8)

### Geometric parameters (Å, º)

**Table:**

O1—C11	1.243 (2)	C2—H2	0.9500
O2—N4	1.233 (2)	C3—C4	1.367 (3)
O3—N4	1.237 (2)	C3—H3	0.9500
O4—N5	1.235 (2)	C4—C5	1.398 (3)
O5—N5	1.235 (2)	C4—H4	0.9500
O6—N6	1.224 (2)	C6—C7	1.396 (3)
O7—N6	1.222 (2)	C7—C8	1.371 (3)
N1—C5	1.351 (2)	C7—H7	0.9500
N1—C1	1.357 (2)	C8—C9	1.392 (3)
N1—H1N	0.92 (2)	C8—H8	0.9500
N2—C5	1.356 (2)	C9—C10	1.372 (3)
N2—C6	1.394 (2)	C9—H9	0.9500
N2—H2N	0.87 (2)	C10—H10	0.9500
N3—C6	1.333 (2)	C11—C16	1.455 (3)
N3—C10	1.345 (2)	C11—C12	1.456 (2)
N4—C12	1.454 (2)	C12—C13	1.369 (2)
N5—C14	1.444 (2)	C13—C14	1.382 (3)
N6—C16	1.460 (2)	C13—H13	0.9500
C1—C2	1.358 (3)	C14—C15	1.388 (3)
C1—H1	0.9500	C15—C16	1.366 (2)
C2—C3	1.400 (3)	C15—H15	0.9500
			
C5—N1—C1	122.30 (16)	N3—C6—C7	123.52 (17)
C5—N1—H1N	113.4 (14)	N2—C6—C7	118.76 (17)
C1—N1—H1N	124.3 (14)	C8—C7—C6	117.67 (18)
C5—N2—C6	128.13 (16)	C8—C7—H7	121.2
C5—N2—H2N	115.1 (14)	C6—C7—H7	121.2
C6—N2—H2N	115.7 (14)	C7—C8—C9	119.76 (19)
C6—N3—C10	117.47 (16)	C7—C8—H8	120.1
O2—N4—O3	122.87 (16)	C9—C8—H8	120.1
O2—N4—C12	119.43 (15)	C10—C9—C8	118.38 (19)
O3—N4—C12	117.69 (15)	C10—C9—H9	120.8
O4—N5—O5	123.15 (15)	C8—C9—H9	120.8
O4—N5—C14	118.32 (15)	N3—C10—C9	123.21 (19)
O5—N5—C14	118.53 (15)	N3—C10—H10	118.4
O7—N6—O6	123.04 (17)	C9—C10—H10	118.4
O7—N6—C16	118.18 (16)	O1—C11—C16	124.53 (16)
O6—N6—C16	118.66 (16)	O1—C11—C12	124.32 (16)
N1—C1—C2	120.28 (17)	C16—C11—C12	111.13 (15)
N1—C1—H1	119.9	C13—C12—N4	116.22 (16)
C2—C1—H1	119.9	C13—C12—C11	124.81 (16)
C1—C2—C3	118.75 (17)	N4—C12—C11	118.93 (15)
C1—C2—H2	120.6	C12—C13—C14	118.97 (17)
C3—C2—H2	120.6	C12—C13—H13	120.5
C4—C3—C2	120.68 (18)	C14—C13—H13	120.5
C4—C3—H3	119.7	C13—C14—C15	121.12 (16)
C2—C3—H3	119.7	C13—C14—N5	119.48 (16)
C3—C4—C5	119.23 (17)	C15—C14—N5	119.38 (16)
C3—C4—H4	120.4	C16—C15—C14	119.34 (17)
C5—C4—H4	120.4	C16—C15—H15	120.3
N1—C5—N2	120.20 (16)	C14—C15—H15	120.3
N1—C5—C4	118.76 (16)	C15—C16—C11	124.48 (16)
N2—C5—C4	121.04 (16)	C15—C16—N6	115.91 (16)
N3—C6—N2	117.71 (16)	C11—C16—N6	119.59 (15)

### Hydrogen-bond geometry (Å, º)

**Table:**

*D*—H···*A*	*D*—H	H···*A*	*D*···*A*	*D*—H···*A*
N1—H1*N*···N3	0.92 (2)	1.86 (2)	2.618 (2)	139 (2)
N1—H1*N*···O4^i^	0.92 (2)	2.45 (2)	3.056 (2)	123.8 (17)
N2—H2*N*···O1^ii^	0.87 (2)	1.97 (2)	2.756 (2)	149 (2)
N2—H2*N*···O2^ii^	0.87 (2)	2.42 (2)	3.114 (2)	136.6 (18)

Symmetry codes: (i) *x*, −*y*+3/2, *z*−1/2; (ii) *x*, −*y*+3/2, *z*+1/2.
